# Cytokine Levels in the Serum of Healthy Subjects

**DOI:** 10.1155/2013/434010

**Published:** 2013-03-07

**Authors:** Giulio Kleiner, Annalisa Marcuzzi, Valentina Zanin, Lorenzo Monasta, Giorgio Zauli

**Affiliations:** Institute for Maternal and Child Health, IRCCS “Burlo Garofolo,” Via dell'Istria, 65/1, 34137 Trieste, Italy

## Abstract

Growing knowledge about the cytokine network response has led to a better comprehension of mechanisms of pathologies and to the development of new treatments with biological drugs, able to block specific molecules of the immune response. Indeed, when the cytokine production is deregulated, diseases often occur. The understanding of the physiological mechanism of the cytokine network would be useful to better comprehend pathological conditions. Moreover, since the immune system and response change their properties with development, differences in patients' age should be taken into account, both in physiological and in pathological conditions. In this study, we analyzed the profile of 48 cytokines and chemokines in the serum of healthy subjects, comparing adults (≥18 years) with young children and children (1–6 and 7–17 years). We found that a certain number of cytokines were not being produced in healthy subjects; others showed a constant serum level amongst the groups. Certain cytokines exhibited a downward or an upward trend with increasing age. The remaining cytokines were up- or downregulated in the group of the children with respect to the other groups. In conclusion, we drew some kinds of guidelines about the physiological production of cytokines and chemokines, underling the difference caused by aging.

## 1. Introduction

Inflammatory response is driven by a complex network of mediators and signaling pathways. For example, cytokines regulating inflammatory response include interleukins that are responsible for communication between white blood cells, chemokines that promote chemotaxis, and interferons that have antiviral effects. Moreover, these molecules are involved in both innate and adaptive immunity, playing a significant physiological role in lymphoid tissue ontogenesis, organogenesis, vasculogenesis, and tissue repair [1, 2]. When the expression of these molecules is chronically altered, diseases often occur. In particular cytokines and chemokines deregulation can support the onset of pathologies linked to chronic inflammation, tumorigenesis, and autoimmunity.

The investigation of a large panel of systemic chemokines and cytokines is important to understand the complete mechanism of immunological changes observed in patients suffering, for example, from inflammatory and autoimmune pathologies. In order to evaluate the significance of cytokines and chemokines modulation in pathological conditions, however, it is necessary to establish the physiological range of these molecules in healthy subjects.

Moreover, even if accumulating evidence suggests that pediatric and adult onset of pathologies often shares similar clinical presentations and basic therapeutic algorithms, we must recognize that children are not miniature adults. Even in the physiological parameter, there are issues specific to children that must be considered in case of disease to ensure prompt diagnosis and an appropriate medical management. Since cytokines and chemokines drive the immune response and the inflammatory processes, it is clear that their expression, at least for some of these molecules, is expected to vary with the growth of the individual, with the immune system achieving gradually a complete maturation [3].

As information on immune cell responses in infancy and early childhood is scarce, we conducted an observational study in which a large cytokine and chemokine panel is analyzed in young children, children, and adults.

The aim of the study is to create guidelines to refer to, especially evaluating if differences in the age of the subjects correspond to differences in the production of cytokines and chemokines.

## 2. Materials and Methods

### 2.1. Subjects

The study was approved by the medical ethics review board of the Institute for Maternal and Child Health, IRCCS “Burlo Garofolo,” Trieste (n.185/08, 19/08/2008). For a child to be eligible, informed consent had to be obtained from parents or caregivers. For ethical reasons we restricted our study population of infants and young children to those who had to undergo a medically indicated peripheral venous blood sampling before elective surgical interventions or within the scope of elective diagnostic procedures. Furthermore, subjects of any age were excluded from the study if they had an acute or chronic infectious disease, any clinically significant disorder, and if they were on any medication with known influence on immunological factors (e.g., corticosteroids). Patients' histories of breast feeding, vaccinations, previous infectious diseases, and allergy were documented but not evaluated as covariates in the study.

Subjects aged more than 18 years were included in one group (≥18). Subjects under the age of 18 years were divided into two groups: young children (1–6 years) and children (7–17 years), considering that in pediatric clinic, diseases that occur before the sixth year of life are defined as early onset pathologies.

### 2.2. Serum Isolation

Whole blood has been collected in a covered test tube without anticoagulants and allowed to clot by leaving it undisturbed at room temperature for 20 minutes. Clot has been then removed by centrifuging at 1500 ×g for 10 minutes in a refrigerated centrifuge. The resulting supernatant was designated serum. Following centrifugation, the liquid component (serum) has been immediately transferred into a clean polypropylene tube using a Pasteur pipette. The samples were maintained at 2–8°C while handling and immediately analyzed, avoiding freeze-thaw cycles because this is detrimental to many serum components.

### 2.3. Determination of Cytokines Release

The analysis of a 48 cytokines and chemokines panel (including IL-1*β*, IL-1ra, IL-2, IL-4, IL-5, IL-6, IL-7, IL-8, IL-9, IL-10, IL-12(p70), IL-13, IL-15, IL-17, Eotaxin, Basic FGF, G-CSF, GM-CSF, IFN-*γ*, IP-10, MCP-1, MIP-1*α*, PDGF-BB, MIP-1*β*, RANTES, TNF-*β*, VEGF, IL-1*α*, IL-2R*α*, IL-3, IL-12(p40), IL-16, IL-18, CTACK, GRO-*α*, HGF, IFN-*α*2, LIF, MCP-3, M-CSF, MIF, MIG, *β*-NGF, SCF, SCGF-*β*, SDF-1*α*, TNF-*α*, TRAIL) was performed on serum samples using a magnetic bead-based multiplex immunoassays (Bio-Plex) (BIO-RAD Laboratories, Milano, Italy) following manufactures' instructions. Data from the reactions were acquired using the Bio-Plex 200 reader, while a digital processor managed data output and the Bio-Plex Manager software returned data as Median Fluorescence Intensity (MFI) and concentration (pg/mL).

### 2.4. Data Analysis

For each set of experiments, values were analyzed by calculating medians and interquartile ranges (IQRs). Box plots were used to show median, IQR, and Tukey whiskers values. The nonparametric Kruskal-Wallis test followed by the Dunn's multiple comparison test was used when appropriate. Probability (*P*) values were calculated on the basis of two-tailed tests. Analysis was performed using GraphPad Prism software version 5.0 (GraphPad Software, Inc., La Jolla, CA, USA). A *P* value of less than 0.05 was considered for statistical significance, unless established by the adjustment for multiple comparisons.

## 3. Results

Blood samples were finally taken from 72 control subjects (Table 1).

Several cytokines (IL-1*β*, IL-5, IL-15, IL-1*α*, IL-3, IL-12(p40), IFN-*α*2, LIF, MCP-3, *β*-NGF, and TNF-*β*) were under the lower limit of detection (LLOD) in all subjects, either because levels were very low or because these molecules are not produced by healthy subjects (Table 2).

IL-1ra, IL-2, IL-7, IL-8, IL-9, IL-10, IL-12(p70), Basic FGF, G-CSF, GM-CSF, IP-10, MCP-1, MIP-1*α*, RANTES, VEGF, IL-16, CTACK, HGF, M-CSF, and MIG did not show any significant difference amongst the groups. The production of these molecules seems to be constant throughout development (Table 3).

### 3.1. Cytokines with Significantly Lower Levels in Adults with respect to Children

**Figure 1:**
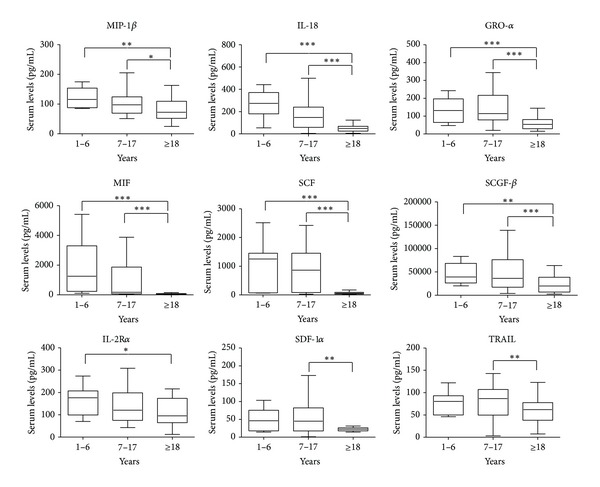
Box plots of serum levels (pg/mL) of cytokines MIP-1*β*, IL-18, GRO-*α*, MIF, SCF, SCGF-*β*, IL-2R*α*, SDF-1*α*, and TRAIL for young children (1–6), children (7–17), and adults (≥18). Whiskers calculated adopting the Tukey method. Outliers not shown. *P < 0.05, **P < 0.01, ***P < 0.001: Kruskal-Wallis test followed by Dunn's multiple comparison test.

Levels of MIP-1*β*, IL-18, GRO-*α*, MIF, SCF, and SCGF-*β* in the adult group were significantly lower if compared to both groups of children. Moreover, IL-2R*α*, SDF-1*α*, and TRAIL showed a similar trend, even if the difference was significant between the adult and only one of the other groups (Figure 1).

### 3.2. Cytokines with Significantly Higher Levels in Adults with respect to Children

**Figure 2:**
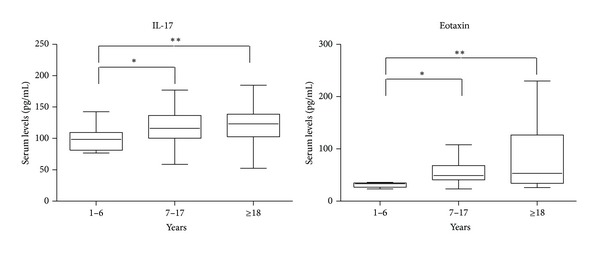
Box plots of serum levels (pg/mL) of cytokines IL-17 and Eotaxin for young children (1–6), children (7–17), and adults (≥18). Whiskers calculated adopting the Tukey method. Outliers not shown. *P < 0.05, **P < 0.01: Kruskal-Wallis test followed by Dunn's multiple comparison test.

IL-17 and Eotaxin showed an increasing trend as age grows. The group of young children showed significantly lower values if compared to both the older children and the adults (Figure 2).

### 3.3. Cytokines Increased or Decreased in the Children (7–17 Years) Group

**Figure 3:**
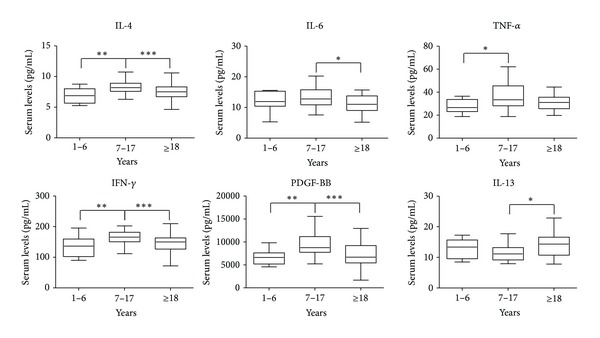
Box plots of serum levels (pg/mL) of cytokines IL-4, IL-6, TNF-*α*, IFN-*γ*, PDGF-BB, and IL-13 for young children (1–6), children (7–17), and adults (≥18). Whiskers calculated adopting the Tukey method. Outliers not shown. *P < 0.05, **P < 0.01, ***P < 0.001: Kruskal-Wallis test followed by Dunn's multiple comparison test.

Cytokines and chemokines IL-4, IL-6, TNF-*α*, IFN-*γ*, PDGF-BB, and IL-13 did not show differences between young children and adults but were all upregulated in children between 7 and 17 years, except IL-13, that was instead downregulated, with respect to the other two groups (Figure 3).

## 4. Discussion

It is well known how important it is to study and identify new inflammatory markers, so as to use them as possible targets for the development of new pharmacological approaches. In order to know if and how much a molecule is deregulated in case of disease, however, we need reference values to be measured in physiological conditions.

Some of these molecules, for example, are supposed to be linked to a specific disease. IL-15 is considered to be the major factor responsible for the immunopathogenesis of celiac disease, and for this reason it has been considered as a possible therapeutic target [4]. IL-7 is known to be an inflammatory mediator associated with arthritic diseases [5], and MIF is a proinflammatory cytokine involved in several inflammatory disorders, including rheumatoid arthritis, inflammatory bowel disease, psoriasis, and multiple sclerosis [6].

Other cytokines and chemokines, involved in several diseases, are already targets of biological drugs [7]. For example, adalimumab, etanercept, and infliximab are agents that inhibit tumor necrosis factor-alpha (TNF-*α*), that is overexpressed in several autoimmune diseases, such as ankylosing spondylitis, psoriatic arthritis, ulcerative colitis, Crohn's disease, and rheumatoid arthritis [8–12]. Anakinra is an interleukin-1 (IL-1) receptor antagonist used for the therapy of several pathologies, such as rheumatoid arthritis, familial Mediterranean fever, gout, juvenile idiopathic arthritis, and even asbestosis [12–16]. Mepolizumab is an antibody that binds IL-5, used in the therapy of hypereosinophilic syndromes and asthma [17].

Several cytokines were not found in the sera of healthy subjects or were below levels of detection. Some of these molecules are distinctive of a pathological situation or of an inflammatory response. Both IL-1*α* and IL-1*β*, for example, are known to be molecules with a strong proinflammatory role [18], IL-15 has been reported to be elevated in celiac patients in previous studies [19], and IL-5 plays an important role in the pathophysiology of asthma [20].

A decreasing trend has been found for MIP-1*β*, IL-18, GRO-*α*, MIF, SCF, SCGF-*β*, IL-2R*α*, SDF-1*α*, and TRAIL, showing that these molecules are mainly produced during childhood and youth with respect to adulthood.

On the contrary, IL-17 and Eotaxin showed an increasing trend, correlated with the age.

Other cytokines (IL-4, IL-6, IL-13, IFN-*γ*, PDGF-BB, and TNF-*α*) showed more complex profiles, with a marked difference in the production in the children group (7–18 years), probably caused by the physiological changes that occur during adolescence. Similar findings were described in other studies [3].

It must be taken into account that this work was conducted on subjects' serum, and thus the levels we found refer to mature and circulating cytokines and chemokines. Moreover, no clear explanation exists to understand how sex, sex hormones, and/or chromosomes affect the immune system and why the female/male ratio is high in several autoimmune and autoinflammatory diseases [21, 22]. The production of cytokines showed negligible and nonsignificant differences in any group when investigated in relation to the sex (data not shown).

In conclusion, even though the findings must be confirmed by further studies with larger cohorts of patients, guidelines were drawn to assist in evaluating the levels of cytokines and chemokines in the serum of healthy subjects of different age, in order to better comprehend the production of these molecules in physiological conditions.

## Figures and Tables

**Table 1:** Characteristics of the sample (*n* = 72).

Group	Frequency	Age	Sex
		(median; min/max)	
1–6 years	7	3; 1/6	M = 1, F = 6
7–17 years	30	11.6; 7/17	M = 14, F = 16
≥18 years	35	36; 21/86	M = 9, F = 26

Total	72	17; 1/86	M = 24, F = 48

**Table 2:** Cytokines and chemokines not detected in any group, either because they are under the lower limit of detection (LLOD, pg/mL) or because they are not produced, and LLOD.

Cytokine	LLOD
IL-1*β*	3.2
IL-5	3.1
IL-15	2.1
IL-1*α*	1.4
IL-3	12
IL-12(p40)	41
IFN-*α*2	307
LIF	12
MCP-3	79
*β*-NGF	1
TNF-*β*	1.5

**Table 3:** Cytokines and chemokines showing an unchanged production amongst groups.

Cytokine	Group 1–6 years	Group 7–17 years	Group ≥18 years
IL-1ra	139.2 (92–185.4)	169.2 (134.7–203.6)	129.6 (101.2–172)
IL-2	10.5 (9.4–12.2)	13.8 (10.2–19.2)	14 (9.4–15.9)
IL-7	12.1 (10.3–14.4)	13.6 (10.9–20)	13.5 (11–17.1)
IL-8	30.9 (23.7–32)	32.6 (28.2–39)	29.3 (24.4–35.9)
IL-9	17.6 (10.9–26.8)	24.6 (20.2–30.5)	23.3 (16.3–31.3)
IL-10	11.4 (9.5–12.8)	11.3 (8.9–13.7)	12.6 (8.5–16.7)
IL-12(p70)	44.3 (39.8–64.1)	34.5 (23.2–48.2)	34.8 (19.6–56.3)
Basic FGF	33.9 (30.8–39.5)	40.1 (35.7–49.3)	41.7 (33.2–49.5)
G-CSF	36.2 (30.3–49.9)	43.8 (39.3–54)	45.5 (34–53.6)
GM-CSF	78.8 (74.6–83)	97.4 (30.1–138.4)	38.3 (26.3–63.8)
IP-10	674.5 (375.4–795.9)	525.8 (387.8–848.9)	576.2 (368.5–808.5)
MCP-1	35.9 (25.6–62)	52 (26.5–77.9)	41.5 (20.1–78.9)
MIP-1*α*	7.3 (6.6–8.1)	7.4 (6.3–8.2)	7.1 (6.2–9)
RANTES	5753 (4890–6201)	5420 (4889–6091)	5839 (5147–6089)
VEGF	95.6 (81.4–169.3)	73.3 (46–126)	61.6 (32–118.9)
IL-16	151 (110.1–205.7)	155.7 (92.2–213.2)	105.6 (93–166)
CTACK	459 (238.5–549.9)	384.4 (268.2–506.2)	335.9 (190.9–468.6)
HGF	464.5 (270.1–689.2)	389.4 (308.3–506.4)	319.7 (196.6–477.9)
M-CSF	10.8 (8.2–26.9)	12.2 (9.1–17)	9.9 (8.6–13.1)
MIG	354.5 (306.2–393.4)	380.1 (167.4–548.8)	278.2 (138.7–539.9)

Values are expressed as pg/mL, median (IQR); differences amongst groups are not statistically significant (*P* > 0.05) after a Kruskal-Wallis test followed by Dunn's multiple comparison test.

## References

[1] Wong MM, Fish EN (2003). Chemokines: attractive mediators of the immune response. *Seminars in Immunology*.

[2] Raman D, Sobolik-Delmaire T, Richmond A (2011). Chemokines in health and disease. *Experimental Cell Research*.

[3] Härtel C, Adam N, Strunk T, Temming P, Müller-Steinhardt M, Schultz C (2005). Cytokine responses correlate differentially with age in infancy and early childhood. *Clinical and Experimental Immunology*.

[4] Benahmed M, Meresse B, Arnulf B (2007). Inhibition of TGF-*β* signaling by IL-15: a new role for IL-15 in the loss of immune homeostasis in celiac disease. *Gastroenterology*.

[5] Hartgring SAY, Bijlsma JWJ, Lafeber FPJG, Van Roon JAG (2006). Interleukin-7 induced immunopathology in arthritis. *Annals of the Rheumatic Diseases*.

[6] Alam A, Pal C, Goyal M (2011). Synthesis and bio-evaluation of human macrophage migration inhibitory factor inhibitor to develop anti-inflammatory agent. *Bioorganic & Medicinal Chemistry*.

[7] Garin A, Proudfoot AEI (2011). Chemokines as targets for therapy. *Experimental Cell Research*.

[8] Braun J, Baraliakos X, Brandt J (2005). Persistent clinical response to the anti-TNF-*α* antibody infliximab in patients with ankylosing spondylitis over 3 years. *Rheumatology*.

[9] Rutgeerts P, Sandborn WJ, Feagan BG (2005). Infliximab for induction and maintenance therapy for ulcerative colitis. *New England Journal of Medicine*.

[10] Woolacott N, Bravo Vergel Y, Hawkins N (2006). Etanercept and infliximab for the treatment of psoriatic arthritis: a systematic review and economic evaluation. *Health Technology Assessment*.

[11] Keating GM, Perry CM (2002). Infliximab: an updated review of its use in Crohn’s disease and rheumatoid arthritis. *BioDrugs*.

[12] Rubbert-Roth A (2012). Assessing the safety of biologic agents in patients with rheumatoid arthritis. *Rheumatology*.

[13] Calligaris L, Marchetti F, Tommasini A, Ventura A (2008). The efficacy of anakinra in an adolescent with colchicine-resistant familial Mediterranean fever. *European Journal of Pediatrics*.

[14] So A, De Smedt T, Revaz S, Tschopp J (2007). A pilot study of IL-1 inhibition by anakinra in acute gout. *Arthritis Research and Therapy*.

[15] Fitzgerald AA, LeClercq SA, Yan A, Homik JE, Dinarello CA (2005). Rapid responses to anakinra in patients with refractory adult-onset Still’s disease. *Arthritis and Rheumatism*.

[16] Dostert C, Pétrilli V, Van Bruggen R, Steele C, Mossman BT, Tschopp J (2008). Innate immune activation through Nalp3 inflammasome sensing of asbestos and silica. *Science*.

[17] Busse WW, Ring J, Huss-Marp J, Kahn JE (2010). A review of treatment with mepolizumab, an anti-IL-5 mAb, in hypereosinophilic syndromes and asthma. *Journal of Allergy and Clinical Immunology*.

[18] Goldbach-Mansky R (2012). Immunology in clinic review series, focus on autoinflammatory diseases: update on monogenic autoinflammatory diseases: the role of interleukin (IL)-1 and an emerging role for cytokines beyond IL-1. *Clinical & Experimental Immunology*.

[19] Zanzi D, Stefanile R, Santagata S (2011). IL-15 interferes with suppressive activity of intestinal regulatory T cells expanded in celiac disease. *American Journal of Gastroenterology*.

[20] Takatsu K (2011). Interleukin-5 and IL-5 receptor in health and diseases. *Proceedings of the Japan Academy B*.

[21] McCombe PA, Greer JM, Mackay IR (2009). Sexual dimorphism in autoimmune disease. *Current Molecular Medicine*.

[22] Lleo A, Battezzati PM, Selmi C, Gershwin ME, Podda M (2008). Is autoimmunity a matter of sex?. *Autoimmunity Reviews*.

